# Factors Affecting the Reinstatement of the Japanese Dental Hygienist: A Japanese Dental Hygienist Survey Conducted in 2019

**DOI:** 10.3390/ijerph18042049

**Published:** 2021-02-19

**Authors:** Ayako Okada, Yoshiaki Nomura, Yuki Ohara, Yuko Yamamoto, Noriyasu Hosoya, Nobuhiro Hanada, Noriko Takei

**Affiliations:** 1 Department of Translational Research, Tsurumi University School of Dental Medicine, Yokohama 230-5801, Japan; okada-a@tsurumi-u.ac.jp (A.O.); hanada-n@tsurumi-u.ac.jp (N.H.); 2 Department of Operative Dentistry, Tsurumi University School of Dental Medicine, Yokohama 230-5801, Japan; 3 Japan Dental Hygienists’ Association, Tokyo 169-0071, Japan; yohara@tmig.or.jp (Y.O.); nori@pm-ms.tepm.jp (N.T.); 4 Tokyo Metropolitan Institute of Gerontology, Tokyo 173-0015, Japan; 5 Department of Endodontology, Tsurumi University School of Dental Medicine, Yokohama 230-5801, Japan; yamamoto-y@tsurumi-u.ac.jp (Y.Y.); hosoya-n@tsurumi-u.ac.jp (N.H.)

**Keywords:** dental hygienist, reinstatement, working environment

## Abstract

There is a shortage of dental hygienists for dental clinics in Japan. An ideal solution would be for dormant dental hygienists to re-enter the workforce. In this study, we identified the obstacles preventing these dental hygienists from re-entering the workforce. The Japan Dental Hygienists’ Association surveyed all 16,113 members about their working conditions. Among the 101 items in the questionnaire, 11 items specifically targeted the reasons why dental hygienists leave their jobs. Among 8780 responses (54.5%), 1063 were from members who had resigned from jobs as dental hygienists. Three hundred and fifty-five (34.4%) answered “Yes” to the question “I would like to return to work if conditions are satisfactory.” The most frequent cause for leaving a job was” Childbirth and child-rearing” (16.9%). “Working status/working hours” (15.7%) was the main obstacle to re-entry. Furthermore, dental hygienists with intentions to re-enter decided to leave their jobs most frequently because of “Childbirth and child-rearing” and “Working status/working hours.” Improvement of the social support system for dental hygienists is required to ensure a sufficient supply of dental health services.

## 1. Introduction

Japan is a super-aging society with the proportion of people aged over 65 years being 28.4%, while the working-age population (15–64 years old) accounts for 59.5% [1]. The proportion of working-age individuals is predicted to decrease even further with retirement and the low birthrate. To address this issue, the Japanese government is introducing reforms to the working environment. Recovering the decreasing birthrate and improving women’s working environment are the main pillars of this reform. Child-rearing support systems have not been sufficient in Japan. The number of childcare facilities and kindergartens is far from sufficient [2]. Moreover, gender and income inequality remain [3].

Japanese law declares that the main task of dental hygienists is the improvement of oral health nationwide. The scope of the work of dental hygienists is oral care and health guidance under the supervision of a dentist, cleaning teeth, the mechanical removal of deposits on the tooth surface over the gingival margin, and assistance with dental treatment. Their dental treatment duties have expanded. There is a strong and increasing demand for oral healthcare in Japan, particularly for older adults. Pneumonia is one of the most common causes of death in Japan, and the crude death rate has been increasing. According to a demographics survey conducted in Japan, aspiration pneumonia resulted in 40,385 deaths in 2019, the sixth most common cause of death [4]. Several studies have shown that the maintenance of oral health and function has noticeable preventive effects against pneumonia, aspiration pneumonia [5,6,7,8], and influenza [9]. Previously, home-based dental care by dental hygienists alone was not allowed. Dentists had to accompany dental hygienists conducting dental care for older adults or handicapped person who could not attend dental clinics. The law of dental hygienists was amended to meet the demands of Japan’s super-aging society. Dental hygienists can perform home-based dental care without a dentist once per week under the Japanese medical insurance and long-term care insurance system.

In addition, perioperative dental care has been introduced to the Japanese medical insurance system. Perioperative oral care management is an effective way to reduce disease complications and hospitalization costs. Many hospitals do not employ dental hygienists. Dentists or dental hygienists from private dental clinics visit hospitals for the oral care management of hospitalized patients. However, this scheme has not yet been thoroughly addressed.

The role of dental hygienists is vital for the prevention of these diseases. However, the number of dental hygienists per head of population is about 1 in 1000. The dental hygienist workforce is not sufficient to provide dental health services under the Japanese national medical insurance system. According to the national survey of Japan, 132,629 dental hygienists out of approximately 200,000 subjects who had licenses worked in 2018 [10]. The remaining 40% were not working in their field of expertise.

In Japan, dental services are sustained mainly by private dental clinics. As of 2018, there were 68,500 dental clinics in Japan [11]. A total of 120,068 (90.5%) dental hygienists included part-time employees working in dental clinics [10]. The average number of dental hygienists per clinic was 1.6. There are turning points in a women’s life—marriage, childbirth, and child-rearing—and the majority of dental hygienists in Japan are female. Working benefit systems are not usually utilized for dental hygienists, because private clinics do not have good benefit and welfare systems. This leads dental hygienists to have a career break. Promoting the re-employment of those who have left their dental hygienist career will increase the number of providers of dental care services.

A survey of the working conditions of dental hygienists is conducted by the Japan Dental Hygienists’ Association every five years. This survey includes items to investigate why dental hygienists leave the professional field. This study aims to identify the obstacles faced by dental hygienists in re-entering their profession and propose countermeasures for supporting re-entry by statistical modeling.

## 2. Materials and Methods

### 2.1. Survey Method

The Japan Dental Hygienists’ Association has been surveying the Employment Status of Japanese dental hygienists every 5 years since 1981. Questionnaires were distributed to all members of the Japan Dental Hygienists’ Association on 16 October 2019 by postage-paid mail with a return envelope. The questionnaires received before the 11 November were used for the analysis. The questionnaire analyzed in this study is provided as supplemental material).

### 2.2. Questionnaire

The questionnaire consisted of 101 items concerning demographic factors, employment situation, job description, and willingness to work.

There were ten specific items targeted at dental hygienists who were leaving their jobs as dental hygienists. In this study, we analyzed these ten specific items.

These items are as follows:

(1) Why did you decide to leave the workplace where you were last working as a dental hygienist? Select all that apply and mark the best answer separately.

1. Dissatisfaction with work content, 2. Payment/welfare, 3. Relationship with the manager, 4. Relationships with seniors, 5. Relationships with colleagues, 6. Working status/working hours, 7. Long working hours/overwork, 8. Family matters, 9. Job transfer of family member, 10. Marriage, 11. Childbirth/child-rearing, 12. Nursing care for family members, 13. Personal health problem, 14. To improve the level of work content, 15. Inadequate training program, 16. Limits of personal skills, 17. The job was not rewarding, 18. Interest in the different fields, 19. Others

(2) What do you think should have been improved about your workplace where you were working last? Select all that apply and mark the best answer separately.

1. Improvement of working conditions (base up, regular pay raise, etc.), 2. Reduction of workload, 3. Interpersonal relationships at the workplace, 4. Reduction of working hours, 5. Taking a vacation, 6. Enhancement of child-rearing support, 7. Enhancement of nursing care support, 8. Evaluation of expertise, qualifications, etc., 9. Enriching opportunities to improve personal skills, through education, training, etc., 10. Introduction of various work styles and working hours, 11. Enhancement of the medical safety system, 12. Ensuring employment stability, 13. Enrichment of employee benefits, 14. Nothing in particular, 15. Other.

(3) How long has it been since you left your job as a dental hygienist?
(4) Do you want to start working as a dental hygienist again? Select one.

1. I want to re-enter as soon as possible, 2. I want to re-enter if conditions are satisfied, 3. I do not intend to, 4. I do not know.

(5) Do you want to start working again at the same workplace where you were last working as a dental hygienist? Select one.

1. I want to re-enter as soon as possible, 2. I want to re-enter if conditions are satisfied, 3. I do not intend to 4. I do not know.

(6) If you were to start working again, how would you find a workplace? Multiple choice.

1. Labor agency, 2. Job magazines, 3. Recommendation from a friend/acquaintance, 4. Recommendation from a dental hygienist school, 5. Internet, 6. Free referral by the Dental Association and Dental Hygienist Association, 7. Human resource dispatch companies, 8. Other

(7) (1) Are there any obstacles that are making it difficult for you to start working again as a dental hygienist?

1. Yes, 2. No

(2) What are these obstacles? Multiple choice.

1. Salary, 2. Working hours, 3. My skills, 4. No consultation, 5. Aging, 6. Others

(8) Where do you want to start working again as a dental hygienist? Multiple choice.

1. Clinic, 2. Hospital/university hospital, 3. Clinic for special needs dentistry, 4. Administration, 5. Dental hygienist education and training institution, 6. Clinic attached to the company, 7. Companies/business establishments, 8. Dental health examination/health activity organization, 9. Social welfare facility, 10. Long-term care insurance facilities, etc. (Please mark the relevant facilities with a circle), 11. Regional comprehensive support center, etc., 12. Research institute, 13. Other.

(9) What are the areas of your interest? Multiple choice.

1. Community health, 2. Home-based dental care (management guidance for in-home care, home-visit oral hygiene management, etc.), 3. Geriatric dentistry, 4. Special needs dentistry, 5. Occupational dental health, 6. Dental hygienist education and training institution, 7. Nothing in particular, 8. Other.

(10) Would you want to participate in a lecture-based course for re-entry?

1. Yes, 2. No.

(11) What kind of lecture-based courses would you want to attend? Multiple choice and choose the best answer.

1. Professional oral care techniques, 2. Scaling and route planning, 3. Dental caries prevention and control, 4. Dental health guidance for individuals, 5. Dental health guidance for group, 6. Handling of dental materials, chairside assistant, 7. Chairside assistant techniques, such as snap impression, polishing of filling materials, rubber dam, etc., 8. Counseling skills, 9. Dysphagia rehabilitation, 10. Myofunctional therapy, 11. Nursing care skills, 12. Other.

The questionnaire is available in Japanese from the Japan Dental Hygienists’ Association [10].

### 2.3. Statistical Analysis

Descriptive statistics and cross-tabulations with age groups were summarized for the questions presented above. Chi-square tests were used to find out the statistical significance. A logit model of log-linear analysis was used to identify statistically significant cells. To visualize the relations, a correspondence analysis was conducted. The results are expressed as biplots. SPSS Statistics Ver 24.0 (IBM, Tokyo, Japan) was used for the analysis.

### 2.4. Ethics Approval and Consent to Participate

Informed written consent was obtained from all participants. This study was approved by the Ethics Committee of the Tsurumi University School of Dental Medicine (approval number: 1837) and conducted in accordance with the Declaration of Helsinki.

## 3. Results

The Japan Dental Hygienists’ Association distributed the questionnaires to 16,113 of its members, and 8780 responded (collection rate: 54.5%). Among them, there were 1063 dormant dental hygienists. In this study, the data analysis included 1063 dental hygienists. Their mean age was 52.36 ± 13.06 years. They were all women.

Table 1 shows a cross-tabulation of the specific questionnaire answered by the dental hygienists who were leaving their jobs. They were classified into age groups of ≤29, 30–39, 40–49, 50–59, and 60–70 years. Among 851 dental hygienists, 351 (34.4%) answered that “They would like to return to work if conditions were satisfied.” Approximately 40% of those in the 30–39 year age group were willing to re-enter the workforce (as soon as possible and if conditions are satisfied).

The main reasons for leaving a job were “Childbirth/child-rearing” (16.9%) and “Relationship with the manager” (15.7%). Leaving due to “Childbirth/child-rearing” was a statistically significant factor for the age groups younger than 49. For those in their 20s and 40s, “Childbirth/child-rearing” and “Relationship with the manager” were the main reasons for leaving a job. For those in their 30s, “Childbirth/child-rearing” (40.6%) was the most frequent reason for leaving a job. For those in their 50s, the most frequent reason for leaving a job was “Interest in a different field” (18.2%). For those in their 60s, the most frequent reason for leaving a job was “Personal health” (16.4%). For those in their 70s, the most frequent reason for leaving a job was “Family matters” (18.1%).

Regarding the item about factors that needed to be improved in the last workplace environment, 285 dental hygienists (28.1%) answered “Interpersonal relationships at the workplace,” which was the most common response, followed by “Improvement of salary (base up, regular pay raise, etc.,)” (25.5%). These answers were statistically significant factors for individuals in all age groups except for those in their 70s. For those aged below 40, the most frequent request was “Interpersonal relationships at the workplace.” For those in their 50s, “Improvement of working conditions (base up, regular pay raise, etc.)” (28.2%) and “Interpersonal relationships at the workplace” (28.2%) were the most common answers. For those in their 60s, “Improvement of the working conditions (base up, regular pay raise, etc.,)” was the most frequent response. For those in their 70s, “Evaluation of expertise, qualification, etc.,” (19.7%) was the most frequent response.

The main obstacle to working again was “Working hours” (58.1%). For respondents in their 20s, “My skills” (75.0%) was a major obstacle. For those in their 30s and 40s, “Working hours” was a major obstacle. For those in their 50s and 60s, “Aging” presented a major obstacle. For those in their 70s, “Working hours” and “Aging” were major obstacles.

Approximately 48% of subjects stated that they wish to participate in an educational or upskill workshop to aid in workplace re-entry. “Counseling skills” was the program that attracted the greatest level of interest (70.0%). “Dysphagia rehabilitation” was a highly requested program for all age groups except participants aged in their 20s. For those younger than 39, the “Scaling and route planning” program was frequently of interest. This was a statistically significant answer for respondents in their 20s, 30s, and 40s. For age groups older than 40, “Counseling skills” was frequently of interest.

In terms of finding a new workplace, approximately 70% of subjects stated that they would use a “Labor agency.” For all generations, “Labor agency” was the most popular answer to this question. For age groups younger than 30,” the number of subjects who answered “Internet” was notable.

A cross-tabulation showing participants’ willingness to re-enter the workforce versus items related to leaving jobs and re-entry is shown in Table 2.

The main reason why dental hygienists who wished to re-enter the workforce had left their jobs was “Childbirth/child-rearing” (17.0%). This reason was statistically significant related to the statement “I want to re-enter if conditions are satisfied.”

For the question on improvements that should be made to their last workplace environment, 28.0% of subjects answered: “Interpersonal relationships at the workplace.” This item was statistically significantly related to the response “I want to re-enter if conditions are satisfied.” The response “I want to re-enter as soon as possible” was statistically significantly related to the answers “Interpersonal relationships at workplace,” “Evaluation of expertise, qualifications, etc.,” “Enhancement of medical safety system,” “Ensuring employment stability,” and “Enrichment of employee benefits.”

In terms of obstacles to working again, 57.9% of subjects selected “Working hours.” This item was identified as the major obstacle for subjects who answered “I want to reenter if conditions are satisfied.” For the subjects who answered “I want to reenter as soon as possible,” “My skills” was identified as the major obstacle.

Many subjects stated that they would be interested in participating in two lecture courses: “Dysphagia rehabilitation” (70.3%) and “Myofunctional therapy” (61.7%). Many of the subjects who answered “I want to re-enter as soon as possible” and “I want to re-enter if conditions are satisfied” selected these two lecture courses.

To find a new workplace, 70.1% of subjects stated that they would use a “Labor agency.” The answers “Recommendation from a friend/acquaintance” and “Internet” were statistically significantly related to the response “I want to re-enter as soon as possible.”

**Figure 1:**
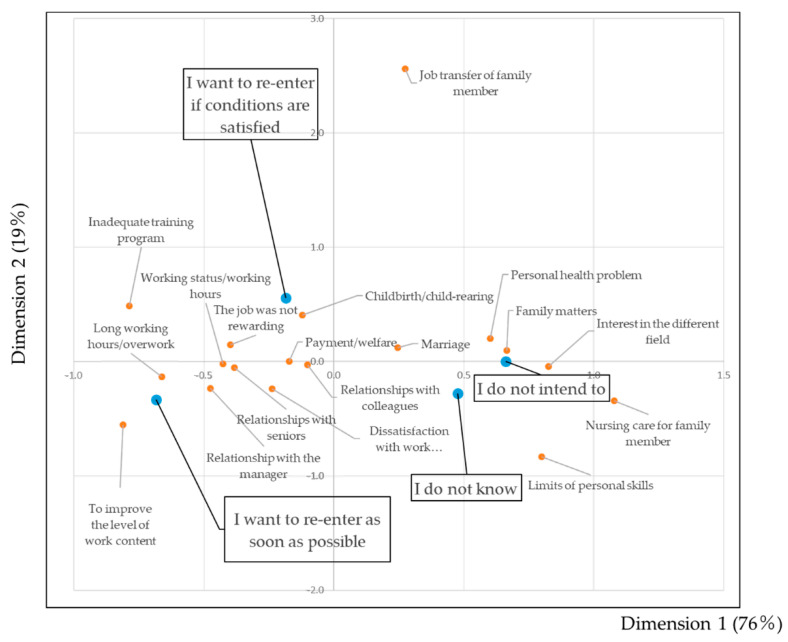
Biplot diagram showing the willingness to re-enter and the reasons for leaving jobs. The blue circles indicate the level of willingness to re-enter: “I want to re-enter as soon as possible,” “I want to re-enter if conditions are satisfied,” “I do not intend to,” and “I do not know.” The red circles show 18 reasons for leaving jobs. The closer the items are to each other, the higher the association between items.

The cross-tabulation of the items investigated in this study against age groups was illustrated graphically by correspondence analysis. Figure 1 shows the biplot of the willingness to re-enter and reasons for leaving jobs. “Working status/working hours” is located near to subjects who answered “I want to re-enter as soon as possible.” The item “Childbirth/child-rearing” is located near to “I want to re-enter if conditions are satisfied.” “Family matters” and “Personal health” are in close proximity to “I do not intend to.”

**Figure 2:**
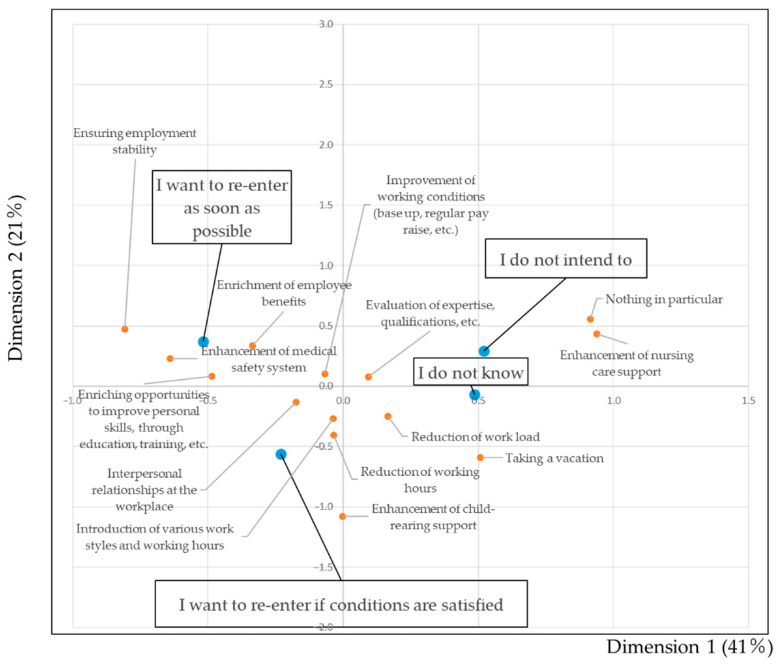
Biplot showing the willingness to re-enter and the need for improvement of the workplace environment. The blue circles show the willingness to re-enter. The red circles show 14 items related to improvement of the workplace environment. The closer the items are, the greater the association between items.

Figure 2 shows a biplot of the willingness to re-enter and the need for improvement of the workplace environment. “Enrichment of employee benefits” is located near to the item “I want to re-enter as soon as possible.” “Reduction of the working load” is located near to the item “I want to reenter if conditions are satisfied.” “Evaluation of expertise, qualifications, etc.,” and “Enhancement of nursing care support” are in close proximity to “I do not intend to.”

## 4. Discussion

The Japan Dental Hygienists’ Association surveys the working conditions of dental hygienists every five years. In this survey, dental hygienists who had left their jobs were included. A report of this survey was presented on the homepage of the Japan Dental Hygienists’ Association [12]. One of the aims of this survey is to support the re-entry of dormant dental hygienists into the workplace. Therefore, the questionnaire contained items concerning factors associated with a leaving job, factors that would support workplace re-entry, and factors associated with improving the workplace environment. However, this report only presents descriptive statistics. A more in-depth analysis including statistical modeling is indispensable to address obstacles to the willingness to re-enter the workforce and to determine the effects of factors on the willingness to re-enter. To address this issue, we applied a log-linear analysis and correspondence analysis to identify the statistically significant factors related to the willingness to re-enter the workforce.

By Japanese dental hygienist law, only women can be licensed dental hygienists. The law was amended in 2015, and since then, men have also been allowed to be licensed as dental hygienists. However, the number of dental hygienists is still at a low level.

More than approximately 40% of dental hygienists surveyed stated that they do not wish to reenter the dental hygienist workforce. The social position of dental hygienists needs to improve and should be reflect by salaries that are commensurate with the work involved. On the other hand, around 40% of dormant dental hygienists stated that they would like to re-enter the workforce. Encouraging dormant dental hygienists to re-enter the workforce is an effective strategy to resolve the supply–demand imbalance. Many dental hygienists who stated that they would like to re-enter feel that there are obstacles to re-entry. Therefore, analyzing the reasons for leaving jobs, obstacles to reentry, and ways to improve the working environment is essential, especially to encourage dental hygienists who wish to reenter the workforce immediately.

The major reason reported for leaving a job was “Childbirth/child-rearing.” These respondents stated that they would like to re-enter the workforce if conditions are improved. The log-linear analysis indicated that “Childbirth/child-rearing” was a statistically significant factor for the younger generation (those aged less than 49 years old). In addition, this item was statistically significantly related to the response “I want to reenter if conditions are satisfied.” The correspondence analysis revealed that “Childbirth/child-rearing” is the most important factor that needs to be addressed to entice dormant dental hygienists to return to work.

“Relationship with the manager” was the second most common reason given for leaving a job. Some dental hygienists stated that they had left jobs due to human relationships with seniors or colleagues. More than half of them wished to re-enter.

In terms of improving the working environment, “Improvement of working condition (base up, regular pay raise, etc.,)” and “Interpersonal relationships in the workplace” had high response rates. The log-linear analysis indicated that the item “Improvement of working conditions (base up, regular pay raise, etc.,)” was a statistically significant response for all generations, except for participants in their 70s. Under the Japanese medical insurance system, each dental practice gives fixed pay. Japanese dental hygienist law regulates the practice of dental hygienists, requiring the instruction of a dentist. The salary of a dental hygienist depends on the interests of the dental office [13]. Further study on the cost-effectiveness of the employment of dental hygienists is necessary. The log-linear analysis indicated that the item “Interpersonal relationships at the workplace” was a statistically significant factor for all generations, except for respondents in their 70s. Moreover, this item was found to be statistically significantly associated with the response “I want to re-enter if conditions are satisfied.” This indicates that good human relationships are the basis of a good working environment. In Japan, the dental service supply mainly depends on private dental clinics, and most of them operate on a small scale. Most of the dental clinics are managed by dentists. It is hard to improve human relationships in private dental clinics because of the limited resources available. One solution is to implement systematic construction of the community through a dental association or dental hygienist association. However, the improvement of human relationships is a complicated task. Further study on improving human relationships is necessary.

When focused on respondents in their 30s, “Enhancement of child-rearing support” and “Introduction of various work style and working hours” were shown to be statistically significant factors. Considering that a frequent reason for leaving a job was “Child-birth/child-rearing,” flexible work arrangements are needed. Unfortunately, most dental clinics in Japan operate on a small scale and are privately managed. The number of dental hygienists is not sufficient. It is currently hard to introduce a flexible working system. Therefore, a social support system is necessary to resolve this issue. The Japanese government has been developing policy measures to improve the working environment for women. Several points need to be improved, including working hours. These factors are not unique to Japan. Similar problems occur in other parts of the world [14,15,16].

Frequent obstacles to re-entry were “Working hours,” “My skills,” and “salary.” For respondents under the age of 50, more than 70% of dental hygienists recognized working hours as being an obstacle. This result has been found in previous studies in Japan [13,14,15,16]. Most Japanese hygienists work in private dental clinics. Many of these clinics are open until 8:00 or 9:00 pm. Some of them are also open on weekends and holidays. Given the situation, dental hygienists may have complaints about the working hours.

“My skills” was also cited as a frequent obstacle. For respondents in their 20s, 75% recognized their skills as being an obstacle. For respondents in their 60s, 32.4% recognized skills as being an obstacle. Moreover, for the subjects who answered “I want to reenter as soon as possible,” “My skills” was a statistically significant response. Many of the subjects stated that they would like to participate in a lecture-based course for reentry. Many of the subjects who answered “I want to re-enter as soon as possible” and “I want to re-enter if conditions are satisfied” selected two lecture courses as being of interest: “Dysphagia rehabilitation” and “Myofunctional therapy.” The results showed a different tendency to previous surveys [17,18]. In particular, a survey of the working environment of Japanese dental hygienists for the fiscal year 2009 reported that participates were interested in ”Scaling and route planning” more than “Myofunctional therapy” or “Dysphagia rehabilitation.” According to the Report on Public Health Administration and Services [4], 1.0% of dental hygienists were working in welfare facilities for the elderly. The number of dental hygienists working in welfare facilities for older people has been increasing year by year: 0.2% in 2010, 0.3% in 2012, 0.4 in 2014, and 0.8% in 2016. Since Japan is a super-aging society, it is necessary for dental hygienists to be aware of the demands of oral health management for older adults [19]. This may reflect the need for dental hygienists to conduct home-based dental care for older adults.

More than 40% of the subjects stated that they would be interested in courses on scaling root plaining and professional oral care techniques. More than 77% of subjects in their 20s and more than half of those in their 40s gave this response. Dental hygienists may recognize these fundamental skills as being a must for working as dental professionals. On the contrary, “limits of personal skills” was not stated as a major reason for leaving a job. Removing anxiety related to skills may promote re-entry. Further improvement of the skill-up system is necessary. Respondents in the younger age group (≤29) stated that they would be interested in receiving a lecture on “Enriching opportunities to improve personal skills, through education, training, etc.” Most dental hygienist schools in Japan involve a 3-year diploma program. In most cases, working as a dental hygienist involves long working hours. There are limited opportunities to participate in postgraduate education or training programs.

There are several limitations to this study. First, the study population was limited to the members of the Japan Dental Hygienists Association. Second, the data were collected from individuals who self-enrolled in the study. Third, the data were self-reported, and the subjects’ actual behaviors were not observed. In addition, not all subjects answered all of the questions.

## 5. Conclusions

Dental clinics in Japan are experiencing a shortage of dental hygienists. Reintroducing dormant dental hygienists into the workforce would be an effective strategy to resolve the supply–demand imbalance. For the re-entry of dormant dental hygienists, the working environment needs to be improved. The introduction of a flexible working system and training programs focused on re-entry as well as the improvement of human relationships may be key factors.

## Figures and Tables

**Table 1:** Cross-tabulation of the items investigated in this study against age groups.

Age	20s	30s	40s	50s	60s	70s	Total	*p*-Value
Do you want to start working as a dental hygienist again?								
I want to re-enter as soon as possible	9 (21.4%)	12 (7.0%)	7 (3.7%)	9 (3.2%)	3 (1.1%)	0 (0.0%)	40 (3.9%)	<0.001
I want to re-enter if conditions are satisfied	26 (61.9%)	107 (62.6%)	90 (48.1%)	90 (32.3%)	36 (13.2%)	2 (2.9%)	351 (34.4%)	
I do not intend to	2 (4.8%)	22 (12.9%)	49 (26.2%)	111 (39.8%)	195 (71.4%)	65 (94.2%)	444 (43.5%)	
I do not know	5 (11.9%)	30 (17.5%)	41 (21.9%)	69 (24.7%)	39 (14.3%)	2 (2.9%)	186 (18.2%)	
Why did you decide to leave the workplace where you were last working as a dental hygienist?								
Dissatisfaction with work content	6 (14.0%)	27 (15.4%)	25 (13.2%)	23 (8.0%)	13 (4.6%)	4 (5.6%)	98 (9.4%)	0.001
Payment/welfare	6 (14%)	28 (16.0%)	33 (17.5%)	47 (16.4%)	20 (7.1%)	1 (1.4%)	135 (12.9%)	<0.001
Relationship with the manager	15 (34.9%)	34 (19.4%)	42 (22.2%)	45 (15.7%)	27 (9.6%)	1 (1.4%)	164 (15.7%)	<0.001
Relationships with seniors	9 (20.9%)	21 (12.0%)	23 (12.2%)	10 (3.5%)	2 (0.7%)	0 (0.0%)	65 (6.2%)	<0.001
Relationships with colleagues	3 (7.0%)	13 (7.4%)	23 (12.2%)	26 (9.1%)	15 (5.4%)	1 (1.4%)	81 (7.8%)	0.031
Working status/working hours	8 (18.6%)	24 (13.7%)	39 (20.6%)	33 (11.5%)	19 (6.8%)	1 (1.4%)	124 (11.9%)	<0.001
Long working hours/overwork	5 (11.6%)	21 (12.0%)	21 (11.1%)	15 (5.2%)	10 (3.6%)	2 (2.8%)	74 (7.1%)	0.001
Family matters	1 (2.3%)	17 (9.7%)	24 (12.7%)	43 (15.0%)	39 (13.9%)	13 (18.1%)	137 (13.1%)	0.116
Job transfer of family member	2 (4.7%)	12 (6.9%)	0 (0.0%)	6 (2.1%)	3 (1.1%)	3 (4.2%)	26 (2.5%)	<0.001
Marriage	2 (4.7%)	27 (15.4%)	30 (15.9%) *	36 (12.6%)	22 (7.9%)	4 (5.6%)	121 (11.6%)	0.012
Childbirth/child-rearing	15 (34.9%) *	71 (40.6%) *	42 (22.2%) *	33 (11.5%)	12 (4.3%)	4 (5.6%)	177 (16.9%)	<0.001
Nursing care for family member	1 (2.3%)	3 (1.7%) *	7 (3.7%)	26 (9.1%)	34 (12.1%)	7 (9.7%)	78 (7.5%)	<0.001
Personal health	9 (20.9%)	24 (13.7%)	30 (15.9%)	31 (10.8%)	46 (16.4%)	11 (15.3%)	151 (14.4%)	0.327
To improve the level of work content	6 (14.0%) *	8 (4.6%)	7 (3.7%)	5 (1.7%)	3 (1.1%)	0 (0.0%)	29 (2.8%)	<0.001
Inadequate training program	4 (9.3%)	8 (4.6%)	8 (4.2%)	6 (2.1%)	3 (1.1%)	0 (0.0%)	29 (2.8%)	0.007
Limits of personal skills	4 (9.3%)	14 (8.0%)	14 (7.4%)	16 (5.6%)	10 (3.6%)	5 (6.9%)	63 (6.0%)	0.323
The job was not rewarding	3 (7.0%)	14 (8.0%)	14 (7.4%)	13 (4.5%)	6 (2.1%)	1 (1.4%)	51 (4.9%)	0.022
Interest in a different field	1 (2.3%)	17 (9.7%)	19 (10.1%)	52 (18.2%) *	21 (7.5%)	4 (5.6%)	114 (10.9%)	<0.001
Others	7 (16.3%) *	31 (17.7%) *	44 (23.3%) *	74 (25.9%)	123 (43.9%)	35 (48.6%)	314 (30.0%)	<0.001
What do you think should have been improved about your workplace where you were working last?								
Improvement of working conditions (base up, regular pay raise, etc.,)	9 (21.4%) *	56 (32.7%) *	48 (26.1%) *	79 (28.2%) *	62 (23.0%) *	4 (6.1%)	258 (25.5%)	0.001
Reduction of work load	10 (23.8%)	36 (21.1%)	29 (15.8%)	29 (10.4%)	36 (13.3%)	7 (10.6%)	147 (14.5%)	0.015
Interpersonal relationships at the workplace	22 (52.4%) *	61 (35.7%) *	74 (40.2%) *	79 (28.2%) *	48 (17.8%) *	1 (1.5%)	285 (28.1%)	<0.001
Reduction of working hours	10 (23.8%) *	40 (23.4%) *	31 (16.8%)	36 (12.9%)	22 (8.1%)	6 (9.1%)	145 (14.3%)	<0.001
Taking a vacation	10 (23.8%) *	43 (25.1%) *	37 (20.1%)	57 (20.4%) *	28 (10.4%)	6 (9.1%)	181 (17.9%)	<0.001
Enhancement of child-rearing support	6 (14.3%)	33 (19.3%) *	15 (8.2%)	11 (3.9%)	7 (2.6%)	3 (4.5%)	75 (7.4%)	<0.001
Enhancement of nursing care support	0 (0.0%)	0 (0.0%) *	3 (1.6%)	8 (2.9%)	11 (4.1%)	4 (6.1%)	26 (2.6%)	0.034
Evaluation of expertise, qualifications, etc.	4 (9.5%)	29 (17.0%)	33 (17.9%)	41 (14.6%)	54 (20.0%)	13 (19.7%)	174 (17.2%)	0.431
Enriching opportunities to improve personal skills through education, training, etc.	11 (26.2%) *	29 (17.0%)	27 (14.7%)	36 (12.9%)	35 (13.0%)	6 (9.1%)	144 (14.2%)	0.145
Introduction of various work styles and working hours	3 (7.1%)	25 (14.6%) *	17 (9.2%)	24 (8.6%)	14 (5.2%)	1 (1.5%)	84 (8.3%)	0.005
Enhancement of the medical safety system	5 (11.9%)	20 (11.7%)	15 (8.2%)	24 (8.6%)	11 (4.1%)	2 (3.0%)	77 (7.6%)	0.03
Ensuring employment stability	3 (7.1%)	19 (11.1%)	14 (7.6%)	25 (8.9%)	22 (8.1%)	2 (3.0%)	85 (8.4%)	0.484
Enrichment of employee benefits	6 (14.3%)	32 (18.7%)	27 (14.7%)	49 (17.5%)	34 (12.6%)	7 (10.6%)	155 (15.3%)	0.378
Nothing in particular	5 (11.9%) *	26 (15.2%) *	34 (18.5%) *	67 (23.9%) *	86 (31.9%)	29 (43.9%)	247 (24.4%)	<0.001
Other	4 (9.5%)	15 (8.8%)	18 (9.8%)	17 (6.1%)	24 (8.9%)	4 (6.1%)	82 (8.1%)	0.69
Are there any obstacles that are making it difficult for you to start working again as a dental hygienist?								
Yes	24 (72.7%)	106 (89.1%)	84 (87.5%)	88 (90.7%)	34 (87.2%)	1 (50.0%)	337 (87.3%)	0.07
What are these obstacles?								
Salary	8 (33.3%)	32 (30.5%)	23 (27.4%)	18 (20.9%)	3 (8.8%)	0 (0.0%)	84 (25.1%)	0.125
Working hours	16 (66.7%)	81 (77.1%)	59 (70.2%)	24 (27.9%)	13 (38.2%)	1 (100%)	194 (58.1%)	<0.001
My skills	18 (75.0%)	43 (41.0%)	41 (48.8%)	47 (54.7%)	11 (32.4%)	0 (0.0%)	160 (47.9%)	0.011
No consultation	4 (16.7%)	5 (4.8%)	6 (7.1%)	9 (10.5%)	4 (11.8%)	0 (0.0%)	28 (8.4%)	0.393
Aging	0 (0.0%) *	4 (3.8%) *	16 (19.0%)	51 (59.3%)	21 (61.8%)	1 (100%)	93 (27.8%)	<0.001
Others	4 (16.7%)	37 (35.2%)	25 (29.8%)	23 (26.7%)	7 (20.6%)	0 (0.0%)	96 (28.7%)	0.348
Would you want to participate in a lecture-based course for re-entry?								
Yes	27 (62.8%)	108 (63.5%)	116 (61.1%)	152 (53.1%)	85 (31.3%)	9 (13.4%)	497 (48.3%)	<0.001
What kind of lecture courses would you want to attend?								
Professional oral care techniques	7 (25.9%)	62 (57.4%) *	66 (56.9%) *	63 (41.4%)	26 (30.6%)	1 (11.1%)	225 (45.3%)	<0.001
Scaling and route planning	21 (77.8%) *	72 (66.7%) *	62 (53.4%) *	40 (26.3%)	12 (14.1%)	0 (0.0%)	207 (41.6%)	<0.001
Dental caries prevention and control	11 (40.7%)	38 (35.2%)	42 (36.2%)	23 (15.1%)	7 (8.2%)	0 (0.0%)	121 (24.3%)	<0.001
Dental health guidance for individuals	10 (37.0%)	58 (53.7%) *	53 (45.7%)	58 (38.2%)	25 (29.4%)	0 (0.0%)	204 (41.0%)	0.001
Dental health guidance for groups	5 (18.5%)	38 (35.2%)	39 (33.6%)	55 (36.2%)	24 (28.2%)	2 (22.2%)	163 (32.8%)	0.433
Handling of dental materials, chairside assistant	9 (33.3%)	38 (35.2%)	39 (33.6%)	21 (13.8%)	3 (3.5%)	0 (0.0%)	110 (22.1%)	<0.001
Chairside assistant tasks, such as snap impression, polishing of filling materials, rubber dam, etc.	10 (37.0%)	39 (36.1%)	35 (30.2%)	17 (11.2%)	4 (4.7%)	0 (0.0%)	105 (21.1%)	<0.001
Nursing care	10 (37.0%)	44 (40.7%)	48 (41.4%)	58 (38.2%)	41 (48.2%)	5 (55.6%)	206 (41.4%)	0.659
Counseling skills	13 (48.1%)	68 (63.0%)	85 (73.3%)	117 (77%)	58 (68.2%)	7 (77.8%)	348 (70.0%)	0.022
Dysphagia rehabilitation	8 (29.6%)	66 (61.1%)	77 (66.4%)	102 (67.1%)	49 (57.6%)	5 (55.6%)	307 (61.8%)	0.009
Myofunctional therapy	7 (25.9%)	31 (28.7%)	56 (48.3%)	71 (46.7%)	36 (42.4%)	5 (55.6%)	206 (41.4%)	0.012
Other	1 (3.7%)	6 (5.6%)	12 (10.3%)	12 (7.9%)	6 (7.1%)	0 (0.0%)	37 (7.4%)	0.652
If you were to start working again, how would you find a workplace?								
Labor agency	23 (69.7%)	88 (73.9%)	71 (74.1%)	64 (66.7%)	21 (56.8%)	1 (11.1%)	268 (70.1%)	0.349
Job magazines	12 (36.4%)	43 (36.1%)	29 (30.2%)	30 (31.3%)	11 (29.7%)	0 (0.0%)	125 (32.6%)	0.803
Recommendation from a friend/acquaintance	15 (45.5%)	61 (51.3%)	40 (41.7%)	43 (44.8%)	21 (56.8%)	1 (11.1%)	181 (47.3%)	0.610
Recommendation from dental hygienist school	9 (27.3%)	22 (18.5%)	11 (11.5%)	9 (9.4%)	2 (5.4%)	0 (0.0%)	53 (13.8%)	0.039
Internet	21 (63.6%)	72 (60.5%)	42 (43.8%)	36 (37.5%)	5 (13.5%)	0 (0.0%)	176 (46.1%)	<0.001
Free referral by the Dental Association and Dental Hygienist Association	9 (27.3%)	27 (22.7%)	22 (22.9%)	37 (38.5%)	9 (24.3%)	1 (11.1%)	105 (27.4%)	0.110
Human resource dispatch companies	2 (6.1%)	5 (4.2%)	15 (15.6%)	9 (9.4%)	1 (2.7%)	0 (0.0%)	32 (8.4%)	0.044
Other	0 (0.0%)	3 (2.5%)	3 (3.1%)	5 (5.2%)	1 (2.7%)	0 (0.0%)	12 (3.1%)	0.752

* Statistically significant as determined by the log-linear analysis.

**Table 2:** Characteristics of the participants involved in this study by classification of willingness to re-enter.

	I Want to Re-Enter As Soon As Possible	I Want to Re-Enter if Conditions Are Satisfied	I Do Not Intend to Re-Enter	I Do Not Know	Total	*p*-Value
Why did you decide to leave the workplace where you were last working as a dental hygienist?						
Dissatisfaction with work content	6 (15%)	41 (11.6%)	30 (6.8%)	20 (10.6%)	97 (9.5%)	0.063
Payment/welfare	7 (17.5%)	63 (17.9%)	36 (8.2%)	28 (14.9%)	134 (13.1%)	<0.001
Relationship with the manager	12 (30.0%)	76 (21.6%)	43 (9.8%)	31 (16.5%)	162 (15.9%)	<0.001
Relationships with seniors	4 (10%)	31 (8.8%)	16 (3.6%)	12 (6.4%)	63 (6.2%)	0.017
Relationships with colleagues	4 (10%)	34 (9.7%)	26 (5.9%)	15 (8.0%)	79 (7.7%)	0.239
Working status/working hours	8 (20%)	61 (17.3%)	33 (7.5%)	21 (11.2%)	123 (12%)	<0.001
Long working hours/overwork	6 (15%)	36 (10.2%)	23 (5.2%)	9 (4.8%)	74 (7.2%)	0.006
Family matters	3 (7.5%)	39 (11.1%)	68 (15.4%)	21 (11.2%)	131 (12.8%)	0.166
Job transfer of family member	0 (0%)	17 (4.8%)	8 (1.8%)	1 (0.5%)	26 (2.5%)	0.006
Marriage	4 (10.0%)	47 (13.4%)	44 (10.0%)	23 (12.2%)	118 (11.6%)	0.5
Childbirth/child-rearing	7 (17.5%)	93 (26.4%) *	41 (9.3%) *	33 (17.6%)	174 (17.0%)	<0.001
Nursing care for family member	1 (2.5%)	19 (5.4%) *	34 (7.7%)*	24 (12.8%)	78 (7.6%)	0.012
Personal health	3 (7.5%)	56 (15.9%)	58 (13.2%)	33 (17.6%)	150 (14.7%)	0.248
To improve the level of work content	3 (7.5%)	13 (3.7%)	7 (1.6%)	5 (2.7%)	28 (2.7%)	0.079
Inadequate training program	2 (5%)	18 (5.1%) *	7 (1.6%)	2 (1.1%)	29 (2.8%)	0.008
Limits of personal skills	2 (5%)	10 (2.8%) *	31 (7.0%)	19 (10.1%)	62 (6.1%)	0.006
The job was not rewarding	3 (7.5%)	27 (7.7%)	12 (2.7%)	9 (4.8%)	51 (5.0%)	0.014
Interest in different field	2 (5.0%)	33 (9.4%)	51 (11.6%)	26 (13.8%)	112 (11.0%)	0.250
Others	11 (27.5%)	88 (25.0%)	157 (35.6%) *	49 (26.1%)	305 (29.9%)	0.006
What do you think should have been improved about your workplace where you were working last?						
Improvement of working conditions (base up, regular pay raise, etc.)	15 (37.5%)	102 (29.1%)	87 (20.5%) *	52 (28.6%)	256 (25.7%)	0.007
Reduction of work load	6 (15.0%)	62 (17.7%)	48 (11.3%)	30 (16.5%)	146 (14.6%)	0.073
Interpersonal relationships at the workplace	15 (37.5%)	128 (36.6%) *	88 (20.7%)	48 (26.4%)	279 (28.0%)	<0.001
Reduction of the working load	6 (15.0%)	69 (19.7%)	43 (10.1%)	25 (13.7%)	143 (14.3%)	0.002
Taking a vacation	4 (10.0%)	78 (22.3%)	64 (15.1%)	35 (19.2%)	181 (18.2%)	0.034
Enhancement of child-rearing support	2 (5.0%)	43 (12.3%)	16 (3.8%)	13 (7.1%)	74 (7.4%)	<0.001
Enhancement of nursing care support	1 (2.5%)	4 (1.1%) *	10 (2.4%)	9 (4.9%)	24 (2.4%)	0.061
Evaluation of expertise, qualifications, etc.	9 (22.5%) *	66 (18.9%) *	64 (15.1%)	36 (19.8%)	175 (17.6%)	0.316
Enriching opportunities to improve personal skills through education, training, etc.	9 (22.5%)	66 (18.9%)	51 (12.0%)	14 (7.7%)	140 (14%)	0.001
Introduction of various work styles and working hours	4 (10.0%)	38 (10.9%)	2 4 (5.6%)	17 (9.3%)	83 (8.3%)	0.062
Enhancement of the medical safety system	6 (15.0%) *	36 (10.3%) *	25 (5.9%)	9 (4.9%)	76 (7.6%)	0.016
Ensuring employment stability	9 (22.5%) *	39 (11.1%)	23 (5.4%)	14 (7.7%)	85 (8.5%)	<0.001
Enrichment of employee benefits	11 (27.5%) *	61 (17.4%)	57 (13.4%)	24 (13.2%)	153 (15.3%)	0.055
Nothing in particular	8 (20.0%)	45 (12.9%) *	136 (32.0%)	53 (29.1%)	242 (24.3%)	<0.001
Other	3 (7.5%)	34 (9.7%)	30 (7.1%)	12 (6.6%)	79 (7.9%)	0.489
Are there any obstacles that are making it difficult for you to start working again as a dental hygienist? (“Yes”)						
Yes	24 (66.7%) *	315 (89.5%)	0	0	339 (87.4%)	<0.001
What are these obstacles?						
Salary	10 (41.7%)	74 (23.8%)			84 (25.1%)	0.052
Working hours	11 (45.8%)	183 (58.8%)			194 (57.9%)	0.214
My skills	13 (54.2%)	147 (47.3%)			160 (47.8%)	0.514
No consultation	4 (16.7%)	24 (7.7%)			28 (8.4%)	0.127
Aging	7 (29.2%)	86 (27.7%)			93 (27.8%)	0.873
Others	8 (33.3%)	89 (28.6%)			97 (29.0%)	0.624
Would you want to attend a lecture-based course for re-entry? (“Yes”)						
Yes	28 (70.0%)	285 (81.7%)	64 (14.7%)	111 (59.7%)	488 (48.3%)	<0.001
What kind of lecture courses would you want to attend?						
Professional oral care techniques	16 (57.1%)	135 (47.4%)	21 (32.8%) *	54 (48.6%)	226 (46.3%)	0.094
Scaling and route planning	14 (50%)	136 (47.7%)	14 (21.9%) *	43 (38.7%)	207 (42.4%)	0.001
Dental caries prevention and control	10 (35.7%)	72 (25.3%)	10 (15.6%)	31 (27.9%)	123 (25.2%)	0.158
Dental health guidance for individuals	13 (46.4%)	125 (43.9%)	17 (26.6%) *	50 (45.0%)	205 (42.0%)	0.063
Dental health guidance for group	11 (39.3%)	95 (33.3%)	15 (23.4%)	41 (36.9%)	162 (33.2%)	0.270
Handling of dental materials, chairside assistant	5 (17.9%)	70 (24.6%)	5 (7.8%) *	31 (27.9%)	111 (22.7%)	0.013
Chairside assistant techniques, e.g., snap impression, polishing of filling materials, rubber dam, etc.	5 (17.9%)	69 (24.2%)	7 (10.9%)	26 (23.4%)	107 (21.9%)	0.122
Counseling skills	14 (50.0%)	116 (40.7%)	28 (43.8%)	47 (42.3%)	205 (42.0%)	0.797
Dysphagia rehabilitation	22 (78.6%)	195 (68.4%)	45 (70.3%)	81 (73.0%)	343 (70.3%)	0.620
Myofunctional therapy	19 (67.9%)	175 (61.4%)	35 (54.7%)	72 (64.9%)	301 (61.7%)	0.520
Nursing care skills	9 (32.1%)	111 (38.9%)	27 (42.2%)	54 (48.6%)	201 (41.2%)	0.249
Other	1 (3.6%)	22 (7.7%)	4 (6.3%)	9 (8.1%)	36 (7.4%)	0.838
If you were to start working again, how would you find a workplace?						
Labor agency	27 (75.1%)	243 (69.61%)			270 (70.1%)	0.502
Job magazines	9 (25.1%)	117 (33.5%)			126 (32.7%)	0.299
Recommendation from a friend/acquaintance	5 (13.9%) *	177 (50.7%)			182 (47.3%)	<0.001
Recommendation from dental hygienist school	2 (5.6%)	51 (14.6%)			53 (13.8%)	0.133
Internet	24 (66.7%) *	154 (44.1%)			178 (46.2%)	0.010
Free referral by the Dental Association and Dental Hygienist Association	7 (19.4%)	98 (28.1%)			105 (27.3%)	0.268
Human resource dispatch companies	4 (11.1%)	28 (81.0%)			32 (8.3%)	0.523
Other	2 (5.6%)	10 (2.9%)			12 (3.1%)	0.376

* Statistically significant cell according to the log-linear analysis.

## Data Availability

Data are available from the corresponding author on reasonable request.

## Supplementary Materials

The following are available online at https://www.mdpi.com/1660-4601/18/4/2049/s1, File 1: Questionnaire analyzed in this study.

## References

[1] Population Estimates, Statistics Bureau, Ministry of Internal Affairs and Communications Japan. https://https://www.stat.go.jp/english/data/jinsui/tsuki/index.html.

[2] For Achievement of the Zero Children Waiting List, Ministry of Internal Affairs and Communication Japan. https://www.mhlw.go.jp/english/policy/children/children-childrearing/dl/150407-03.pdf.

[3] Labour Force Survey, Statistics Bureau, Ministry of Internal Affairs and Communications Japan. https://www.stat.go.jp/data/roudou/sokuhou/tsuki/pdf/gaiyou.pdf.

[4] Vital Statistics, Ministry of Health, Labor and Welfare Japan. https://www.mhlw.go.jp/toukei/saikin/hw/jinkou/kakutei19/dl/11_h7.pdf.

[5] Barnes C.M. (2014). Dental hygiene intervention to prevent nosocomial pneumonias. J. Evid. Based. Dent. Pract..

[6] Yoon M.N., Steele C.M. (2012). Health care professionals’ perspectives on oral care for long-term care residents: Nursing staff, speech-language pathologists and dental hygienists. Gerodontology.

[7] Adachi M., Ishihara K., Abe S., Okuda K. (2007). Professional oral health care by dental hygienists reduced respiratory infections in elderly persons requiring nursing care. Int. J. Dent. Hyg..

[8] Adachi M., Ishihara K., Abe S., Okuda K., Ishikawa T. (2002). Effect of professional oral health care on the elderly living in nursing homes. Oral. Surg. Oral. Med. Oral. Pathol. Oral. Radiol. Endod..

[9] Abe S., Ishihara K., Adachi M., Sasaki H., Tanaka K., Okuda K. (2006). Professional oral care reduces influenza infection in elderly. Arch. Gerontol. Geriatr..

[10] Report on Public Health Administration and Services, Ministry of Health, Labor and Welfare Japan. https://www.mhlw.go.jp/toukei/saikin/hw/eisei/18/dl/kekka2.pdf.

[11] Survey of Medical Institutions, Ministry of Health, Labor and Welfare Japan. https://www.mhlw.go.jp/toukei/saikin/hw/iryosd/19/dl/02sisetu01.pdf.

[12] Survey of the Working Environment of Japanese Dental Hygienists for the Fiscal Year 2019, Japan Dental Hygienists Association. https://www.jdha.or.jp/pdf/aboutdh/r2-dh_hokoku.pdf.

[13] Hopcraft M., McNally C., Ng C., Pek L., Pham T.A., Phoon W.L., Poursoltan P., Yu W. (2008). Attitudes of the Victorian oral health workforce to the employment and scope of practice of dental hygienists. Aust. Dent. J..

[14] Hopcraft M., McNally C., Ng C., Pek L., Pham T.A., Phoon W.L., Poursoltan P., Yu W. (2008). Working practices and job satisfaction of Victorian dental hygienists. Aust. Dent. J..

[15] Gibbons D.E., Corrigan M., Newton J.T. (2001). A national survey of dental hygienists: Working patterns and job satisfaction. Br. Dent. J..

[16] Yavnai N., Bilder L., Sgan-Cohen H., Zini A. (2012). Dental hygienists in Israel: Employment evaluation, job satisfaction, and training implications. J. Dent. Educ..

[17] Nomura Y., Okada A., Yamamoto Y., Kakuta E., Tomonari H., Hosoya N., Hanada N., Yoshida N., Takei N. (2020). Factors Behind Leaving the Job and Rejoining it by the Japanese Dental Hygienist. Open Dent. J..

[18] Usui Y., Miura H. (2015). Workforce re-entry for Japanese unemployed dental hygienists. Int. J. Dent. Hyg..

[19] Kanazawa N. (2014). Prospects and Subjects of Dental Hygienists−Aiming at the Coordination with Medical Care and Elderly Care. Ann. Jpn. Prosthodont. Soc..

