# Supplementary material for: Factors Affecting the Reinstatement of the Japanese Dental Hygienist: A Japanese Dental Hygienist Survey Conducted in 2019
Source: Int J Environ Res Public Health. 2021 Feb 19;18(4):2049. doi: 10.3390/ijerph18042049 (PMC7922012; doi:10.3390/ijerph18042049)
Supplement: Supplementary file 1 [file ijerph-18-02049-s001.pdf]

We would like to ask dental hygienists who are not currently working.

Question 92) Why did you decide to leave the workplace where you were the last working as

1. Dissatisfaction with work content   2. Payment/welfare   3. Relationship with the manager   4. Relationships with seniors   5. Relationships with colleagues   6. Working status/working hours   7. Long working hours/overwork   8. Family matters   9. Job transfer of family member   10. Marriage   11. Childbirth/child-rearing   12. Nursing care for family members   13. Personal health problem   14. To improve the level of work content   15. Inadequate training program   16. Limits of personal skills   17. The job was not rewarding   18. Interest in the different fields   19. Others

a dental hygienist? Select all that apply and mark the best answer separately.

Question 93) What do you think should have been improved about your workplace where

1. Improvement of working conditions (base up, regular pay raise, etc.) 2. Reduction of work load 3. Interpersonal relationships at the workplace 4. Reduction of working hours 5. Taking a vacation 6. Enhancement of child-rearing support 7. Enhancement of nursing care support 8. Evaluation of expertise, qualifications, etc. 9. Enriching opportunities to improve personal skills, through education, training, etc. 10. Introduction of various work styles and working hours 11. Enhancement of medical safety system 12. Ensuring employment stability 13. Enrichment of employee benefits 14. Nothing in particular 15. Other

you were working last? Multiple choice and choose the best answer.

Question 94) How long has it been since you left your job as a dental hygienist?

Approximately years

1. I want to reenter as soon as possible (Please proceed to Question 96.) 2. I want to reenter if conditions are satisfied (Please proceed to Question 96.) 3. I do not intend to (Please proceed to Question 101.) 4. I do not know (Please proceed to Question 101.)

Question 95) Do you want to start working again as a dental hygienist? Select one.

Question 96) Do you want to start working again at the same workplace where you were last

1. I want to reenter as soon as possible 2. I want to reenter if conditions are satisfied 3. I do not intend to 4. I do not know

working as a dental hygienist? Select one.

Question 97) In case you are to start working again, how do you plan to find the workplace? Multiple choice.

1. Labor agency 2. Job magazines 3. Recommendation from a friend/acquaintance 4. Recommendation from dental hygienist school 5. Internet 6. Free referral by Dental Association and Dental Hygienist Association 7. Human resource dispatch companies 8. Other

Question 98) Are there any obstacles that are making it difficult for you to start working again as a dental hygienist?

1. Yes 2. No

Question 98-1) What are these obstacles? Multiple choice.

1. Salary 2. Working hours 3. My skills 4. No consultation 5. Aging 6. Others

Question 99) Where do you want to start working again as a dental hygienist? Multiple choice.

1. Clinic 2. Hospital/university hospital 3. Clinic for special needs dentistry 4. Administration 5. Dental hygienist education and training institution Aging 6. Clinic attached to the company Others 7. Companies/business establishments 8. Dental health examination/health activity organization 9. Social welfare facility 10. Long-term care insurance facilities, etc. (Please mark the relevant facilities with a circle) 11. Regional comprehensive support center, etc. 12. Research institute 13. Other

Question 100) What are the areas of your interest? Multiple choice.

1. Community health 2. Home dental care (management guidance for in-home care, home-visit oral hygiene management, etc.) 3. Geriatric dentistry 4. Special needs dentistry 5. Occupational dental health 6. Dental hygienist education and training institution 7. Nothing in particular 8. Other

Question 101) Do you want a lecture course for re-entry?

1. Community health 2. Home dental care (management guidance for in-home care, home-visit oral hygiene management, etc.) 3. Geriatric dentistry 4. Special needs dentistry 5. Occupational dental health 6. Dental hygienist education and training institution 7. Nothing in particular 8. Other

1. Yes 2. No

↓

Question 101-1) What kind of lecture courses do you want to attend? Multiple choice and

1. Professional oral care techniques 2. Scaling and route planning 3. Dental caries prevention and control 4. Dental health guidance for individuals 5. Dental health guidance for group 6. Handling of dental materials, chairside assistant 7. Chairside assistant such as snap impression, polishing of filling materials, rubber dam, etc. 8. Counseling skill 9. Dysphagia rehabilitation 10. Myofunctional therapy 11. Nursing care skill 12. Other

choose the best answer.
